# Effect of coadministration of omega-3 fatty acids with glimepiride on glycemic control, lipid profile, irisin, and sirtuin-1 in type 2 diabetes mellitus patients: a randomized controlled trial

**DOI:** 10.1186/s12902-023-01511-2

**Published:** 2023-11-25

**Authors:** Rehab H. Werida, Aalaa Ramzy, Youssri Nassief Ebrahim, Maged Wasfy Helmy

**Affiliations:** 1 Clinical Pharmacy & Pharmacy Practice, Faculty of Pharmacy, Damanhour University, Damanhour, 22514 Egypt; 2 Ministry of Health and Population, Damanhour City, Egypt; 3 Internal Medicine and Diabetes Department, Damanhour Medical National Institute, Damanhour City, Egypt; 4 Pharmacology and Toxicology, Faculty of Pharmacy, Damanhour University, Damanhour, 22514 Egypt; 5 Pharmacology and Toxicology, College of Pharmacy, Arab Academy for Science, Technology and Maritime Transport, Abou Keer, Alexandria, Egypt

**Keywords:** Omega-3, Glimepiride, Irisin, Sirtuin-1, T2DM, Atherogenic index

## Abstract

**Background and objective:**

Type 2 diabetes mellitus (T2DM) is caused by insulin resistance or tissue insensitivity to insulin, as well as relative insulin insufficiency. Diabetes that is uncontrolled for an extended period of time is linked to substantial comorbidities and organ damage. The purpose of the current study is to assess the effect of coadministration of omega-3 fatty acids with glimepiride on blood glucose, lipid profile, serum irisin, and sirtuin-1 levels in T2DM patients.

**Methods:**

This clinical trial involved 70 type 2 diabetic patients randomly assigned to glimepiride 3 mg with either omega-3 capsules contained fish oil 1000 mg, 13% of eicosapentaenoic acid (EPA) and 9% docosahexaenoic acid (DHA) (omega-3 group, *n* = 35) or placebo capsules contained corn oil and linoleic acid (control group, *n* = 35) daily for three months. Blood samples were obtained at the start of the study and 12 weeks later for biochemical examination of HbA1c%, FBG, fasting insulin, and lipid profile. In addition, the atherogenic index of plasma (AIP) was calculated. Human enzyme-linked immunosorbent assay (ELISA) kits were utilized for assessing serum irisin and sirtuin-1 levels before and after the intervention.

**Results:**

Compared to the control group, omega-3 fatty acids decreased serum fasting blood glucose (FBG, *p* < 0.001), glycated hemoglobin percent (HbA1C%, *p* < 0.001), total cholesterol (TC, *p* < 0.001), triglycerides (TGs, *p* = 0.006), low density lipoprotein (LDL, *p* = 0.089), and Homeostatic Model Assessment for Insulin Resistance (HOMA-IR, *p* = 0.021) after three months of intervention. However, a significant increase was reported in serum irisin and high density lipoprotein (HDL) between both groups after intervention (*p* = 0.026 and *p* = 0.007, respectively). The atherogenic index of plasma (AIP) increased in the control group but decreased in the omega-3 group, with significant differences between the two groups (*p* < 0.001).

**Conclusion:**

The present study found that supplementing with omega-3 fatty acids might dramatically enhance blood irisin levels, as well as improve glycemic control and lipid profile in type 2 diabetes mellitus patients using glimepiride.

**Trial Registration:**

This study is registered on ClinicalTrials.gov under identifier NCT03917940. (The registration date: April 17, 2019).

## Introduction

Type 2 diabetes mellitus (T2DM) is a chronic metabolic illness in which the patient’s capability to produce sufficient amounts of insulin is diminished or patients display insulin resistance; thus, the capability of insulin to accomplish its normal function becomes disrupted [1]. Physical inactivity and obesity are the foremost risk factors for type 2 diabetes mellitus [1]. The insulin resistance was attributable to an insulin signaling defect that seems to be caused by increased lipid accumulation. Previous studies have proposed a reduction in the number of insulin receptors in muscle, adipose tissue, and liver in obese subjects [2].

Inflammation is recognized as a protective immune response to infections and tissue injury causing the migration of immune cells and plasma proteins to the infection site or tissue damage. Although inflammation is a useful response to the body and may be addressed quickly, uncontrolled inflammatory reactions can result in excessive or long-term tissue damage, leading to the development of acute or chronic inflammatory illnesses [3]. Chronic inflammation, oxidative stress, and decreased mitochondrial activity in skeletal muscle or adipose tissue are all linked to the development of insulin resistance and type 2 diabetes. Consequently, oxidative stress and inflammation reduction, as well as mitochondrial function maintenance, should be therapeutic objectives for insulin resistance and T2DM [4].

Recently, Bostrom et al. [5] have identified irisin, as a bioactive molecule which is known as 'myokine' and could increase energy expenditure by enhanced thermogenesis, promote weight loss, and improve insulin resistance caused by diet.

Previous study has illustrated that inflammatory processes and oxidative stress are suppressed by situin-1 (SIRT1) [6].

Glimepiride, a second-generation sulfonylurea, stimulates insulin release and can be utilized as monotherapy or in combination with other oral hypoglycemic agents or insulin. Glimepiride has a lower risk of hypoglycemia in comparison with other sulfonylureas. It has been shown to have extra-pancreatic effects such as stimulating lipogenesis and glycogenesis [7]. Glimepiride also appears to efficiently control blood glucose levels without weight gain [8].

Several studies in recent years have demonstrated the importance of omega-3 supplementation including docosahexaenoic acid (DHA) and eicosapentaenoic acid (EPA) when treating various diseases. For instance, in subjects with residual hypertriglyceridemia, the addition of omega-3 fatty acids to atorvastatin improved triglycerides (TG) and non-HDL-C levels in comparison with atorvastatin alone [9]. A recent study suggested that fish oil supplementation is effective in reducing inflammation and improving insulin resistance in obese and T2DM patients independent of weight loss [10]. Several studies reported that DHA and EPA could prevent cardiovascular disease through the anti-inflammatory effect [11]. Moreover, Valle Flores et al. found that supplementation with omega-3 fatty acids significantly reduced the concentrations of inflammation markers in patients with chronic kidney disease (CKD) on hemodialysis [12].

The present study aims to assess the effect of coadministration of omega-3 fatty acids and glimepiride as a hypoglycemic drug on the blood levels of irisin, sirtuin-1, glucose homeostasis, and the lipid profile of type 2 diabetic patients.

The primary outcome in this trial was the improvement of FBG, HbA1C %, lipid profile (total cholesterol (TC), triglycerides (TG), HDL-cholesterol (HDL-C), and LDL-cholesterol (LDL-C)), and atherogenic index of plasma (AIP). Secondary outcomes included the change of serum levels of the measured biomarkers irisin and sirtuin-1 after 12 weeks of intervention.

## Patients and methods

### Study design

Patients were randomly allocated to either the omega-3 group, managed by glimepiride 3 mg tablets plus omega 3 capsules [1000 mg fish oil, 13% of eicosapentaenoic acid (EPA) and 9% docosahexaenoic acid (DHA)] daily, or the control group, managed by glimepiride 3 mg plus placebo daily for 12 weeks, in a controlled, single-blind parallel clinical study. Placebo capsules contained corn oil, linoleic acid and were identical in shade and taste with omega 3 fish oil capsules produced by the Arab Company for Gelatin and Pharmaceutical products, El-Amreya, Alexandria, Egypt. Participants had to be willing to participate, be 30–60 years old, had T2DM for at least 2 years, have HbA1C score of more than 7% [13], and have a body mass index of 25 to 35 kg/m2. Patients were instructed to stop using any dietary supplements at least two weeks before the start of the study and for the duration of the intervention. Exclusion criteria included; using omega-3 supplements in the three months before the start of the study, having chronic renal failure, a hepatic disorder, gastrointestinal or inflammatory disease, or a thyroid disorder, being on thiazolidinediones, warfarin, fibrates, or insulin therapy or needing insulin based on a physician's recommendation, having diabetic comorbidities including micro- and macrovascular problems, being pregnant or nursing, and having a history of drug misuse. During the research period, all individuals were advised to maintain their regular levels of physical activity and nutritional intake. All participating patients provided informed consent. The study was authorized by the Research Ethics Committee of the Faculty of Pharmacy at Damanhour University (Ref. no. 1018PP5) and registered on ClinicalTrials.gov as NCT03917940. The study followed CONSORT criteria and was carried out in conformity with the ethics standards outlined in the Declaration of Helsinki in 1964 and its subsequent revisions, or similar ethics standards.

### Patients and biochemical analyses

This research included 80 T2DM patients (31 men and 49 women) randomly assigned in a 1:1 ratio to one of two groups: the control group (glimepiride + placebo) or the omega-3 group (omega-3 fatty acids + glimepiride). Since the randomization was done by a study-independent individual using a computerized random number technique in Microsoft Excel, the patients would be unaware of the group to which they have been allocated. At the beginning of the study, the weight and height of all patients were taken using a measuring scale, and body mass index (BMI) was calculated using the following formula: weight (in kilograms)/(height × height) (in meters) [14].

Blood samples were collected in the morning, after an overnight fast, and centrifuged at 3,000 rpm for 10 min at room temperature for serum separation. The fasting serum samples were stored at –80 °C until analysis.

The enzymatic colorimetric approach was utilized for measuring serum triglycerides [15] and total cholesterol [16]. The precipitation technique [16] was used to determine high-density lipoprotein cholesterol (HDL-C). Low-density lipoprotein cholesterol (LDL-C) was estimated using the Friedewald formula [17]: LDL = [TC—HDL—(TG/5)] if the TG level was less than 400 mg/dL. The homeostasis model assessment (HOMA) online calculator was utilized for calculating insulin resistance (IR) by the following equation: (Fasting insulin, uIU/mL)X (Fasting glucose, mg/dL) / 405 [18]. The log (TG/HDL-C) method was used to calculate the atherogenic index of plasma (AIP) according to Dobiasova and Frohlich [19]. The enzyme-linked immunosorbent assay (ELISA) kit (Sunred Biological Technology, Shanghai, China: Catalogue No.: 201–12-5328 and 201–12-2558, respectively) was utilized for assessing irisin and SIRT1 levels according to the manufacturer’s directions.

The patients' adherence was evaluated using a pill count and was calculated by subtracting the number of pills left in the bottle from the total number of pills supplied, during the patient-scheduled refill. The predicted number of pills consumed by participants was determined by multiplying the daily dose by the number of days since the pills were supplied. The number of pills consumed was then divided by the total number of pills at the start, and the result was multiplied by 100 to calculate the percent of pill count adherence [20]. We classified 95–100% of pills taken during the follow-up session as good adherence while less than 95% of medicine taken was considered poor adherence, hence excluded from the study. Clinical pharmacist interviewed participants weekly and asked about any specific complaints or side effects known to occur with their treatment using safety monitoring checklist.

### Statistical analysis

The sample size was assessed using G*Power software version 3.1.0 (Institut fur Experimentelle Psychologie, Heinrich Heine Universitat, Dusseldorf, Germany). A total sample size of 70 patients was determined to have a power of 95% to notice a medium to large effect size of 0.88 in the measured outcomes. The obtained data were loaded into a computer and analyzed with the IBM SPSS software package version 25.0 (Armonk, NY: IBM Corp.). The Kolmogorov–Smirnov and Shapiro–Wilk tests were utilized to identify the normality of the data. Variables not having a normal distribution are presented as the median (range), and they were analyzed using the Wilcoxon signed ranks test to compare before and after the intervention, and the Mann–Whitney test was used to compare between the two groups. Continuous normal distributed variables were presented as Mean ± Standard Deviation (SD) and were assessed using paired *t*-test, to determine the difference between baseline and three months after treatment, and independent *t*-test, used to compare between groups. Spearman's rank correlation coefficient was utilized to evaluate the correlation between variables. In order to describe the predicted accuracy of different markers, a receiver operating characteristic (ROC) curve was drawn and the area under the ROC curve was determined. All analyses were conducted on an intention-to-treat (ITT) basis. *P* ≤ 0.05 was chosen as the significance level for the reported results.

## Results

**Fig. 1:**
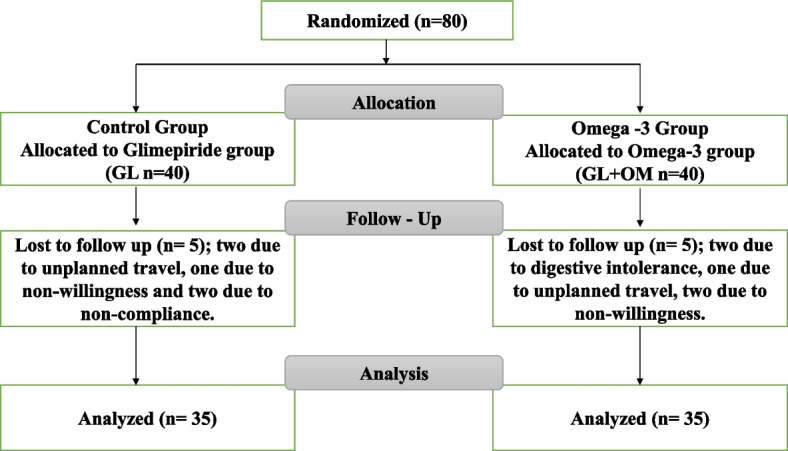
Flow chart of the patients enrollment, randomization, and follow-up during the study

The enrollment and follow-up process of the participants are demonstrated in Fig. 1. A total of 80 patients were recruited initially, but only 70 patients completed the study successfully with no changes to their diet or the level of physical activity throughout the study duration. Ten patients withdrew from the study: two due to digestive intolerance to the supplement, one due to unplanned travel, and two due to non-willingness in omega-3 group, while in control group 5 patients was lost to follow-up; two due to unplanned travel, one due to non-willingness, and two due to non-compliance. Accordingly, 70 patients were included in the final analysis: 35 patients in each group.

Patients’ baseline data and characteristics are summarized in Table 1. The mean age of enrolled patients was 52.42 ± 7.64 years in the control group versus 50.51 ± 8.42 years in the omega-3 group, with no significant difference between both groups. The most common recorded associated diseases in the medical history of participants in the control group compared to the omega-3 group were hypertension (34.3% versus 37.1%, respectively) followed by dyslipidemia (17.1% versus 5.7%, respectively), with no significant difference between both groups as shown in Table 1.

**Table 1:** Baseline characteristics of the studied patients in both groups

	Control group (*n* = 35)	Omega 3 group (*n* = 35)	*P*
Age (year)	52.42 ± 7.64	50.51 ± 8.42	0.323
Gender (Male/Female)	21 (60)/14(40)	17 (48.6)/ 18 (51.4)	0.337
Smoker	8 (22.9)	12 (34.3)	0.290
HTN	12 (34.3)	13 (37.1)	0.803
Dyslipidemia	6 (17.1)	2 (5.7)	0.133
ACEIs	9 (25.7)	8 (22.9)	0.780
ARBs	3 (8.6)	0 (0)	0.080
Beta Blocker	4 (11.4)	8 (22.9)	0.205
CCB	6 (17.1)	4 (11.4)	0.495
Statin	10 (28.6)	5 (14.3)	0.145

Data are presented as Mean ± SD or Frequency (percent) as appropriate

Data analyzed by Chi-square test or independent t-test as appropriate. Statistically significant at *p* ≤ 0.05

*HTN* Hypertension, *CAD* Coronary Artery Diseases, *ACEIs* Angiotensin Converting Enzyme Inhibitors, *ARBs* Angiotensin Receptor Blockers, *CCB* Calcium Channel Blocker

**Fig. 2:**
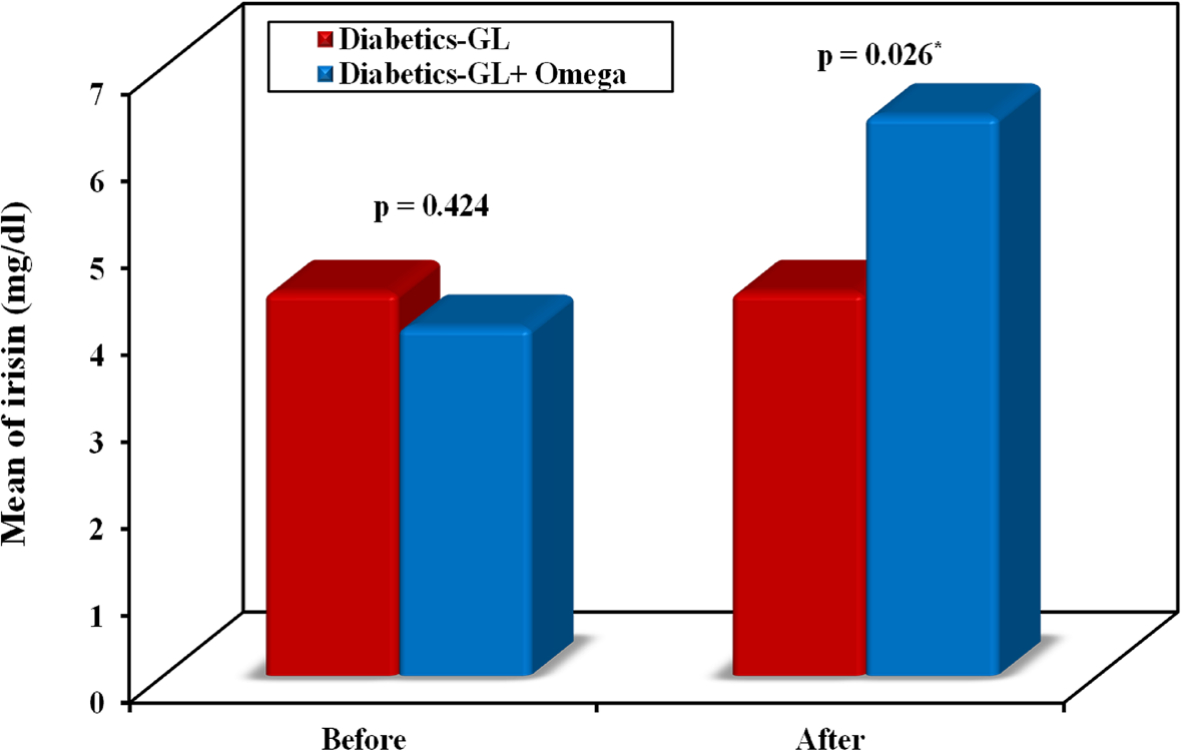
Comparison of irisin level between the two studied groups

**Fig. 3:**
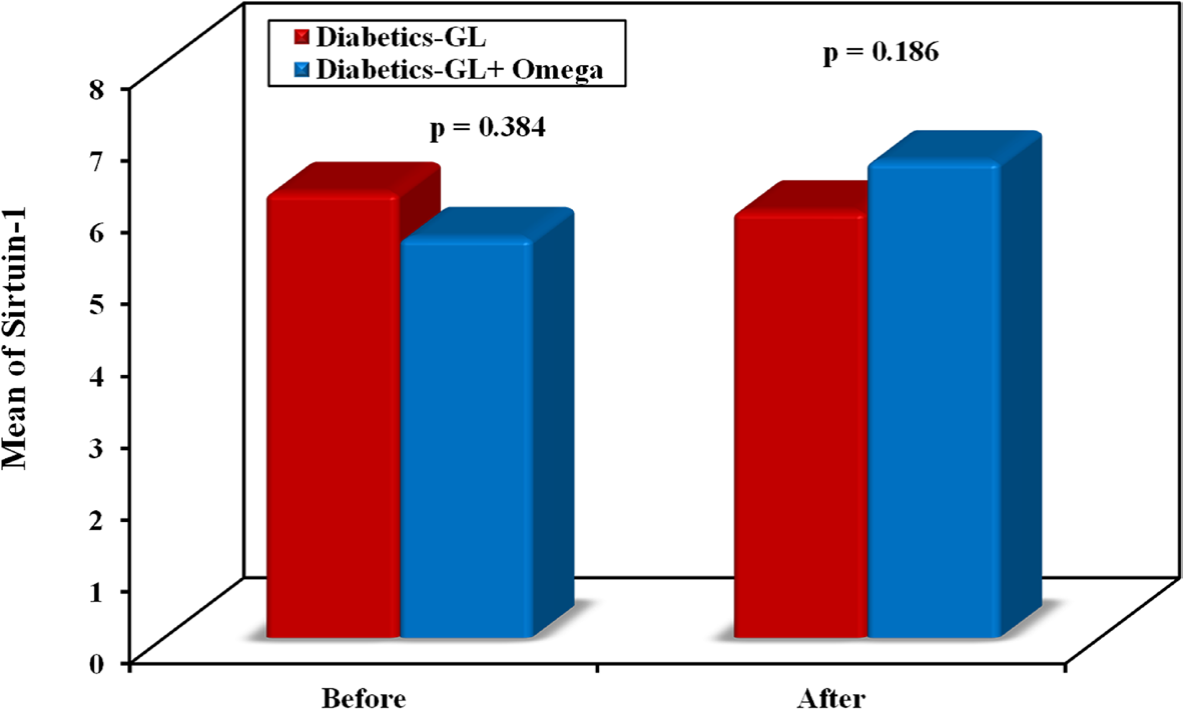
Comparison of situin-1 level between the two studied groups

No significant difference was observed regarding the co-administered medications between both groups as shown in Table 1. There were no statistically significant differences observed in all the baseline characteristics between the two groups except for AIP. At the end of the study, there was a reduction in the levels of FBG [143 (110–350) to 140 (110–320) versus 145(90–214) to 118(70–158), *p* < 0.001], HbA1c % [8.38 ± 0.93 to 8.09 ± 1.04 versus 8.53 ± 1.19 to 6.82 ± 0.84, *p* < 0.001],HOMA-IR [5.3 (3–14.5) to 4.5 (2.8–13.4) versus 4.1 (1.8–11.6) to 3.8 (1.7 – 7), *p* = 0.021], TC [212.1 ± 31.76 to 204.7 ± 32.61 versus 198.0 ± 30.06 to 145.1 ± 25.89, *p* < 0.001], LDL [116.2 ± 13.05 to 111.9 ± 12.35 versus 121.2 ± 18.23 to 105.6 ± 17.50, *p* = 0.089] and TGs [171.5 ± 26.09 to 165.2 ± 23.63 versus 177.0 ± 23.41 to 146.8 ± 29.76, *p* < 0.006] in the patients of the control group in comparison with the patients receiving omega 3 plus glimepiride in omega-3 group, respectively as shown in Table 2. A significant increase in the levels of HDL [47.69 ± 8.79 to 47.57 ± 8.25 versus 43.60 ± 11.79 to 53.94 ± 10.76, *p* = 0.007] and irisin [3.8(0.5–14.5) to 3.9(1.5–13.1) versus 3.5(0.9–20.2) to 4.7 (1.9 – 37.6), *p* = 0.026] was observed in the control group in comparison with the omega-3 group, respectively (Fig. 2). Meanwhile, a non-significant difference was found in the level of sirtuin-1 when comparing both groups despite the significant increase in sirtuin-1 level in the omega-3 group after intervention in comparison with baseline (*p* = 0.04) as shown in Table 2 and Fig. 3. The atherogenic index of plasma (AIP) increased in the control group and decreased in the omega-3 group in comparison with baseline with significant differences between both groups (*p* < 0.001).

**Table 2:** Comparison of clinical features and laboratory investigations between the control group and omega-3 group at baseline and after 12 weeks of intervention and follow-up

	**Control group (*n* = 35)**			**Omega 3 group (*n* = 35)**			**p**_**1**_	**p**_**2**_
	**Before**	**After**	**p**	**Before**	**After**	***p***		
**BMI (kg/m**^**2**^**)**	32.82 ± 2.08	33.15 ± 2.42	t_0_p0.022^*^	32.50 ± 2.45	32.21 ± 2.43	t_0_p0.001^*^	^t^0.558	^t^0.111
**FBG (mg/dl)**	143 (110 – 350)	140 (100 – 320)	^Z^p 0.003^*^	145 (90 – 214)	118 (70 – 158)	^Z^*p* < 0.001^*^	^U^0.605	^U^ < 0.001^*^
**HbA1c %**	8.38 ± 0.93	8.09 ± 1.04	t_0_*p* < 0.001^*^	8.53 ± 1.19	6.82 ± 0.84	t_0_*p* < 0.001^*^	0.548	< 0.001^*^
**Fasting Insulin (mIU/mL)**	13.80 ± 3.18	13.69 ± 2.64	t_0_p0.673	13.15 ± 4.83	14.31 ± 4.32	t_0_p0.025^*^	0.511	0.466
**HOMA-IR**	5.3 (3 – 14.5)	4.5 (2.8 – 13.4)	^Z^p 0.070	4.1 (1.8 – 11.6)	3.8 (1.7 – 7)	^Z^p0.001^*^	^U^0.072	^U^0.021^*^
**TC (mg/dl)**	212.1 ± 31.76	204.7 ± 32.61	t_0_p0.009^*^	198.0 ± 30.06	145.1 ± 25.89	t_0_*p* < 0.001^*^	^t^0.061	^t^ < 0.001^*^
**TG (mg/dl)**	171.5 ± 26.09	165.2 ± 23.63	t_0_p0.001^*^	177.0 ± 23.41	146.8 ± 29.76	t_0_*p* < 0.001^*^	^t^0.353	^t^0.006^*^
**HDL (mg/dl)**	47.69 ± 8.79	47.57 ± 8.25	t_0_p0.816	43.60 ± 11.79	53.94 ± 10.76	t_0_*p* < 0.001^*^	^t^0.105	^t^0.007^*^
**LDL (mg/dl)**	116.2 ± 13.05	111.9 ± 12.35	t_0_p0.001^*^	121.2 ± 18.23	105.6 ± 17.50	t_0_*p* < 0.001^*^	^t^0.194	^t^0.089
**AIP**	0.18 (0– 0.43)	0.19 (0 – 0.37)	^Z^p0.042^*^	0.27 (0.04 – 0.7)	0.10 (0 – 0.28)	^Z^*p* < 0.001^*^	^U^0.046^*^	^U^ < 0.001^*^
**Irisin (mg/dl)**	3.8 (0.5 – 14.5)	3.9 (1.5 – 13.1)	^Z^p0.596	3.5 (0.9 – 20.2)	4.7 (1.9 – 37.6)	^Z^*p* < 0.001^*^	^U^0.424	^U^0.026^*^
**Sirtuin-1**	5.1 (3.8 – 19.1)	5.0 (2.2 – 18.5)	^Z^p0.294	4.7 (3.2 – 17.1)	5.6 (3.3 – 17.5)	^Z^p0.040^*^	^U^0.384	^U^0.186

Data was expressed by using Mean ± SD. if data was normally distributed and Median (Min. – Max.) if data was not normally distributed *SD* Standard deviation

*BMI* Body mass index, *FBG* Fasting blood glucose, *HbA1C %* Glycated hemoglobin, *TC* Total Cholesterol, *TG* Triglycerides, *HDL* High-density Lipoprotein, *LDL* Low-density Lipoprotein, *AIP* Atherogenic Index of plasma

^*^: Statistically significant at *p* ≤ 0.05

t: Independent Student t-test, U: Mann Whitney test

t_0_: Paired Student t-test, Z: Wilcoxon signed ranks test

p: p value for comparing between before and after treatment in each group

p_1_: *p* value for comparing between Control and Omega 3 group before intervention

p_2_: *p* value for comparing between Control and Omega 3 group after intervention

Table 3 shows the impact of baseline characteristics (Gender, age, BMI, HbA1c %, Fasting insulin and AIP) between both groups. On logistic regression analysis and adjustment of the confounders, these confounders showed no significant impact between both groups.

**Table 3:** Logistic regression analysis of the baseline characteristics (Gender, age, BMI, HbA1c %, Fasting insulin and AIP) between both studied groups

	**B**	**S.E**	**Wald**	**df**	***P*-Value**	**Exp(B)**
Gender	-0.329	0.542	0.369	1	0.544	0.720
Age	-0.023	0.032	0.493	1	0.482	0.978
BMI	-0.084	0.114	0.538	1	0.463	0.920
HbA1c %	0.082	0.257	0.102	1	0.749	1.086
Fasting Insulin (mIU/mL)	-0.003	0.066	0.003	1	0.960	0.997
AIP	4.545	2.385	3.631	1	0.057	94.166
Constant	2.382	4.526	0.277	1	0.599	10.822

*BMI* Body mass index, *HbA1C %* Glycated hemoglobin, *AIP* Atherogenic Index of plasma

**Fig. 4:**
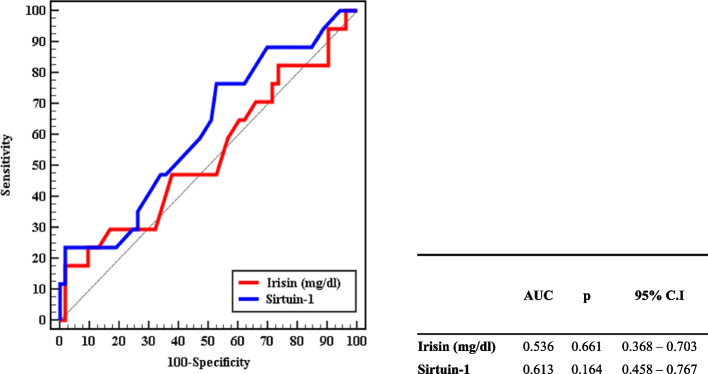
ROC curve for irisin and sirtuin-1 before treatment to predict worsening HOMA-IR (*n* = 17) from improved HOMA-IR (*n* = 53)

Figure 4 shows the validity (AUC, sensitivity, and specificity) of irisin and sirtuin-1 for predicting worsening HOMA-IR (*n* = 17) from improved HOMA-IR (*n* = 53) before treatment. Sirtuin-1 was the most sensitive (AUC = 0.613, *p* = 0.54) followed by irisin (AUC = 0.536, *p* = 0.16).

Regarding tolerability of the used medications during the presented study, there were no reported significant adverse events or complaints among participants in both groups.

## Discussion

The present study aims to assess the effect of coadministration of glimepiride as a hypoglycemic drug and omega-3 fatty acids on the blood levels of irisin, sirtuin-1, glucose homeostasis, and lipid profile of type 2 diabetic patients.

Our results revealed no significant difference in the case of body mass index between both studied groups after 3 months of intervention, which seems in accordance with some previous findings [21].

In our study, we found that FPG, HOMA-IR, and HbA1C levels were reduced in the omega-3 group when compared to the control group, suggesting enhanced insulin sensitivity in the studied diabetic patients. This suggests that the consumption of omega-3 fatty acids supplements could improve glycemic control, FPG, HbA1c %, and HOMA-IR, in T2DM patients, which seems in accordance with some previous findings [21, 22]. Induction of  Peroxisome proliferator-activated receptor gamma coactivator 1-alpha (PGC-1α) by omega-3 fatty acids has been linked to increased glucose transport and insulin sensitivity through glucose transporter 4 (GLUT4) [23]. Omega-3 fatty acids can regulate metabolism and other cell and tissue responses, including adipocyte differentiation and inflammation through activating peroxisome proliferator-activated receptor (PPAR) and inhibiting NFκB. This mechanism might explain the ability of omega-3 fatty acids to increase insulin sensitivity and reduce inflammation [24].

The current study revealed no change in plasma insulin in response to omega 3. Our results agree with those of Mostad et al., [25] who could not find better fasting insulin concentrations after omega-3 supplementation. Furthermore, our results are in line with those reported by Sirtori et al. [26], who found no significant changes in serum insulin levels between the treatment and control groups [26]. On the contrary, Mori et al. reported that both EPA and DHA significantly increased fasting insulin in mildly hyperlipidemic men [27].

The present study indicated a significant reduction in total cholesterol (TC) after omega-3 fatty acids supplementation, which seems in accordance with some previous findings [21].

Previous studies seem to be compatible with our current result, revealing that triglyceride levels and AIP decreased effectively among patients receiving n–3 fatty acids [28, 29]. Some studies reported that triglyceride levels were markedly reduced among patients receiving omega-3 fatty acids in comparison to those receiving a placebo [30]. The present results demonstrated a significant reduction in TG levels and an increase in HDL-C levels (*p* = 0.006 and *p* = 0.007, respectively). Consistent with our results, Kesavulu et al. [31] reported that TG levels significantly decreased, and HDL-C levels significantly increased with the combined treatment with omega-3 fatty acids in diabetic patients. The reported mechanisms by which omega-3 fatty acids reduce TG levels are through the stimulation of fatty acid oxidation, resulting in the reduction of fatty acids substrate for triglyceride synthesis [11]. Furthermore, omega-3 fatty acids can increase plasma lipolytic activity and enhance clearance of plasma TG [32].

Although both groups showed a significant reduction in LDL after 12 weeks, changes in LDL did not differ significantly between groups. Consistent with our results, Eftekhari et al. found that omega-3 fatty acids did not have any significant effect on serum LDL [33]. On the contrary, Agh et al. [34] demonstrated that serum LDL decreased in participants with omega-3 fatty acids supplementation.

The current study indicated that omega-3 supplementation resulted in an increased serum irisin level of type 2 diabetic patients after the intervention between both groups. The results of a study on human rhabdomyosarcoma cells have shown that treatment with omega-3 fatty acids for 24 and 48 h leads to increased expression of the irisin level [23]. Consistent with our results, Agh et al. found that serum irisin levels increased significantly after omega-3 fatty acids supplementation in male patients with coronary artery disease (CAD) [34]. In addition, Ansari et al. [1] revealed that omega-3 supplementation could significantly increase serum irisin levels in type 2 diabetic patients. Treatment with omega-3 fatty acids has been shown to increase the expression of peroxisome proliferator-activated receptor co-activator 1 alpha (PGC-1α) in white adipocytes. PGC-1α increases fatty acid oxidation through the induction of peroxisome proliferator-activated receptor alpha (PPARα). Irisin is induced by PGC-1α expression and increases metabolic rate through activating uncoupling protein 1 (UCP1) in mitochondria [23]. This process increases energy expenditure and improves metabolic parameters including insulin sensitivity [35]. According to a recent study, individuals with chronic renal failure who are receiving hemodialysis may benefit clinically from omega 3 by having higher levels of the protective vascular calcification inhibitors: fetuin-A and osteoprotegerin [36]. Accordingly, physical activity stimulates the transcriptional regulator peroxisome proliferator-activated receptor-coactivator 1 (PGC-1), promoting the expression and proteolytic cleavage of fibronectin type III domain-containing protein 5 (FNDC5) with the release of the irisin in the blood flow. This in turn promotes a browning of white adipose tissue by increasing the expression of mitochondrial uncoupling protein 1 (UCP1) [5].

Another study stated that irisin promotes cell proliferation, as well as insulin production and secretion. Increased circulating irisin has been linked to better glucose tolerance and weight loss [37, 38]. These findings seem to agree with a previous study which displayed that irisin enhanced β cell generation and improved insulin activity in mice [39].

Although serum sirtuin-1 level increased significantly in the omega-3 group, the changes in serum sirtuin-1 level did not differ significantly between groups in the present study. In line with our study, the addition of omega-3 fatty acids in a study on male Sprague–Dawley rats conducted by Son et al. resulted in a relative increase in SIRT1 expression, but this difference was not statistically significant compared to placebo group [40]. Another study found that after six weeks of therapy, ligarglutide dramatically boosted SIRT-1 expression, and this effect persisted for an additional six weeks beyond the end of the regimen. Additionally, they observed a further decline in total cholesterol, ceruloplasmin, fasting glucose, BMI, and HbA1c [41]. Situin-1 (SIRT1) inhibits oxidative stress and inflammatory processes by interfering with nuclear factor kappa-B (NF-κB) signaling by deacetylation of the NF-κB p65 subunit and regulation of its transcriptional activity [6]. SIRT1 has the ability to control PGC-1α deacetylation, which activates peroxisome proliferator-activated receptor-α (PPAR-α), leading to enhanced insulin resistance and increased fatty acid oxidation [4]. Given that SIRT-1 is implicated in the epigenetic regulation of pathogenic pathways in several disorders, including type 2 diabetes [42, 43]. Consistently, given the bidirectional relationship between kidney and cardiovascular events in diabetic patients, antidiabetic therapies with a positive impact on renal and cardiovascular outcomes will play a critical role in reshaping the way diabetes complications are managed [44, 45].

## Conclusion

The current study illustrated that omega-3 fatty acids supplementation could significantly increase serum irisin levels and showed better glycemic control and improved lipid profile and insulin sensitivity in type 2 diabetec patients.

### Study limitations

The current study has some limitations. The main limitation is its relatively small sample size. Other limitations include the intervention duration which was short to understand the real effects of omega-3 fatty acids supplementation and being single blind, controlled study. Hence, further double-blind controlled studies should be carried out on a large scale and for a longer duration to confirm our research findings.

## Data Availability

The data are available from the corresponding author upon reasonable request.
